# Immune checkpoint inhibitor-mediated polymyalgia rheumatica versus primary polymyalgia rheumatica: comparison of disease characteristics and treatment requirement

**DOI:** 10.1093/rheumatology/keae099

**Published:** 2024-02-09

**Authors:** Olof C B Vermeulen, Elisabeth Brouwer, Riemer H J A Slart, Maria Sandovici, Abraham Rutgers, T Jeroen Hilterman, Birgitta Hiddinga, Sjoukje F Oosting, Mathilde Jalving, Albert H de Heij, Daan G Knapen, Geke A P Hospers, Kornelis S M van der Geest

**Affiliations:** Rheumatology and Clinical Immunology, University of Groningen, University Medical Center Groningen, Groningen, The Netherlands; Rheumatology and Clinical Immunology, University of Groningen, University Medical Center Groningen, Groningen, The Netherlands; Nuclear Medicine and Molecular Imaging, University of Groningen, University Medical Center Groningen, Groningen, The Netherlands; Faculty of Science and Technology, Department of Biomedical Photonic Imaging, University of Twente, Enschede, The Netherlands; Rheumatology and Clinical Immunology, University of Groningen, University Medical Center Groningen, Groningen, The Netherlands; Rheumatology and Clinical Immunology, University of Groningen, University Medical Center Groningen, Groningen, The Netherlands; Pulmonology, University of Groningen, University Medical Center Groningen, Groningen, The Netherlands; Pulmonology, University of Groningen, University Medical Center Groningen, Groningen, The Netherlands; Medical Oncology, University of Groningen, University Medical Center Groningen, Groningen, The Netherlands; Medical Oncology, University of Groningen, University Medical Center Groningen, Groningen, The Netherlands; Medical Oncology, University of Groningen, University Medical Center Groningen, Groningen, The Netherlands; Medical Oncology, University of Groningen, University Medical Center Groningen, Groningen, The Netherlands; Medical Oncology, University of Groningen, University Medical Center Groningen, Groningen, The Netherlands; Rheumatology and Clinical Immunology, University of Groningen, University Medical Center Groningen, Groningen, The Netherlands

**Keywords:** polymyalgia rheumatica, immune checkpoint inhibitor, immunotherapy, immune-related adverse events

## Abstract

**Objectives:**

To compare clinical characteristics, imaging findings and treatment requirements of patients with immune checkpoint inhibitor-mediated polymyalgia rheumatica (ICI-PMR) and primary PMR.

**Methods:**

This single centre, retrospective cohort study compared ICI-PMR in patients with cancer (*n* = 15) to patients with primary PMR (*n* = 37). A comparison was made between clinical symptoms, laboratory markers, ultrasonography, ^18^F-FDG-PET/CT findings and treatment requirements related to PMR.

**Results:**

Patients with ICI-PMR less frequently fulfilled the EULAR/ACR classification criteria for PMR (66.7%) than patients with primary PMR (97.3%). Morning stiffness, weight loss and elevation of the ESR were less frequently seen in patients with ICI-PMR. No differences were observed regarding the presence of inflammatory lesions on ultrasound of the shoulders and hips between the two groups. The Leuven and the Leuven/Groningen ^18^F-FDG-PET/CT scores were significantly lower in the ICI-PMR group. Finally, the ICI-PMR group could be managed with lower glucocorticoid doses than the primary PMR group, while this treatment could be discontinued more quickly.

**Conclusion:**

Our findings indicate that ICI-PMR may have a milder course with less intense inflammation than primary PMR. ICI-PMR can be managed with a relatively low glucocorticoid dose. Our study underscores that ICI-PMR should be regarded as a PMR-like syndrome.

Rheumatology key messages

- Inflammation is milder in immune checkpoint inhibitor-mediated polymyalgia rheumatica (ICI-PMR) than in primary PMR.
- ICI-PMR has a low glucocorticoid requirement compared with primary PMR.
- ICI-PMR is a PMR-like syndrome rather than a true form of primary PMR.

## Introduction

The introduction of immune checkpoint inhibitors (ICI) has been a significant breakthrough in the field of oncology. These therapies are based on preventing inhibitory signals mediated by receptors and ligands (PD-1/PD-L1, CTLA4) on cancer cells or on T cells themselves. Currently, the most frequently used ICI therapies are nivolumab (anti-PD-1), pembrolizumab (anti-PD-1), durvalumab (anti-PD-L1), atezolizumab (anti-PD-L1) and ipilimumab (anti-CTLA-4) [1, 2]. ICI therapy has yielded durable responses and, in some cases, long-term survival of patients with a variety of cancers [3]. However, removal of the brake on the immune system can lead to serious immune-related adverse events (irAEs). One of the most common rheumatic irAEs is a polymyalgia rheumatica (PMR)-like syndrome [4].

Primary PMR, occurring in the absence of ICI therapy, is the most common rheumatic inflammatory disease in the elderly. Its incidence peaks at the age of 70–75 years and primary PMR is more prevalent among women. Clinical manifestations of the disease include bilateral pain in the shoulders and pelvic region associated with morning stiffness and constitutional symptoms [5]. Ultrasonography and ^18^F-fluorodeoxyglucose-positron emission tomography/computed tomography (^18^F-FDG-PET/CT) studies have shown that various bursae, tendon sheaths and joints can be inflamed in PMR [5, 6]. Initial treatment of primary PMR consists of oral glucocorticoid (GC) therapy in a daily dose of 12.5–25 mg [7].

Currently, it is debated whether PMR that develops after ICI therapy (ICI-PMR) and primary PMR can be seen as a single nosological entity. Although ICI-PMR may cause typical symptoms in the shoulder and pelvic region, case series have suggested that inflammatory markers in blood are often low in patients with ICI-PMR [8]. Nevertheless, two studies reported use of aggressive immunosuppressive therapy for ICI-PMR [8, 9]. This might be a concern, as such immunosuppressive therapy may potentially impair the anti-tumour effects of ICI therapy. Another report suggested a higher prevalence of peripheral arthritis in patients with ICI-PMR *vs* those with primary PMR [10]. However, studies systematically comparing the clinical, imaging and treatment features of ICI-PMR and primary PMR have not yet been performed. Knowledge about any potential differences may aid in developing improved treatment strategies and better classification of these forms of PMR. The objective of this study was to compare the clinical characteristics, imaging findings and treatment requirement of ICI-PMR *vs* primary PMR.

## Methods

This is a retrospective study of patients diagnosed with ICI-PMR and primary PMR at the Department of Rheumatology and Clinical Immunology of the University Medical Center Groningen (UMCG). In this study, ICI-PMR is defined as PMR-like syndrome occurring in patients who received at least one cycle of ICI, and who demonstrate predominant pain and stiffness in the shoulder and hip girdle, with ultrasonography and/or ^18^F-FDG-PET/CT demonstrating inflammation in at least two sites (that is, two shoulders, two hips, or one shoulder and one hip). An increase of inflammation markers in the blood—erythrocyte sedimentation rate (ESR) or C-reactive protein (CRP)—was not an absolute requirement for the diagnosis.

The study was conducted in accordance with the Declaration of Helsinki and approved by the ethics committee of the University Medical Center Groningen (#202100360 and #201900511). No informed consent was required due to the retrospective nature of the study.

### Patient inclusion

All consecutive patients (*n* = 15) receiving a diagnosis of ICI-PMR between July 2021 and September 2022 were included. Part of these patients have been described elsewhere [11, 12]. For comparison, we have included a well-defined group of patients with primary PMR, in whom the clinical diagnosis of PMR was confirmed after 6 months of follow-up (*n* = 37). The presence of concomitant GCA was ruled out by ^18^F-FDG-PET/CT in these patients. These patients were recruited between December 2010 and May 2020 in the context of a prior study on ^18^F-FDG-PET/CT imaging in PMR, and data on long-term treatment requirement were available [5]. To supplement the ultrasonography data, additional consecutive patients with primary PMR (*n* = 16), in whom the diagnosis was confirmed after 6 months of follow-up, were recruited between May 2021 and May 2022.

### Clinical, laboratory and treatment data

Clinical data in patients with primary PMR and ICI-PMR were collected according to a fixed protocol. The following data were retrieved from electronic patient records: age, sex, symptoms and laboratory data underlying the 2012 European League Against Rheumatism and American College for Rheumatology (EULAR/ACR) criteria and the Chuang criteria for PMR [13, 14]. Furthermore, the presence of fever (>38°C), weight loss (≥2 kilograms) and back pain were collected. Haemoglobin and thrombocyte counts were evaluated. All laboratory data were obtained before glucocorticoid treatment had started, unless stated otherwise. The follow-up period for glucocorticoid treatment was set to a maximum of 990 days after start of glucocorticoid treatment, during this period changes in glucocorticoid doses were noted. Next, the glucocorticoid dose at fixed time points (starting dose, 90 days-, 180 days-, 270 days-, 360 days-, 540 days-, 720 days- and 990 days after the start of glucocorticoid treatment) as well as cumulative doses were determined.

### Ultrasonography

Ultrasonography was performed by experienced rheumatologists (K.S.M.v.d.G. or M.S.) for detection of the following abnormalities: subdeltoid bursitis, bicep tenosynovitis and glenohumeral synovitis in the shoulder region; and coxofemoral synovitis and trochanteric bursitis in the hip region. Measurements were performed on the eSaote MyLab Twice or eSaote MyLab Seven with 3–13 MHz or 6–18 MHz linear probes for the shoulders/hip regions or the 1–8 MHz curvilinear probe for the hip region. Ultrasonographic items of the 2012 EULAR/ACR criteria for PMR were calculated [13].

### ^18^F-FDG-PET/CT

Technical aspects of ^18^F-FDG-PET/CT are provided in Supplementary Methods, available at *Rheumatology* online. Scans were evaluated by a single, experienced nuclear-medicine specialist (R.H.J.A.S.). Evaluation was based on visual grade, where at a specific anatomic region zero points are given when there is no ^18^F-FDG-uptake; one point for less uptake than the liver; two points for equal uptake to the liver; and three points are given for higher uptake than the liver. ^18^F-FDG-uptake was evaluated at the 12 anatomic locations that are used for the Leuven Score; and seven anatomic sites used for the Leuven/Groningen Score [5, 15]. ^18^F-FDG-PET scans performed at the diagnosis of ICI-PMR or primary PMR were performed prior to initiation of glucocorticoid therapy. For comparison, we also included ^18^F-FDG-PET/CT scans from control patients (*n* = 13) with suspected PMR in whom the presence of any rheumatic inflammatory disease (including PMR) was ruled out as part of a prior study [5]. For the ICI-PMR group, ^18^F-FDG-PET/CT scans performed before the initiation of ICI therapy were collected as well.

### Tumour response data

To evaluate the tumour response in the ICI-PMR group, the Response Evaluation Criteria in Solid Tumours 1.1 (RECIST 1.1) as well as the adapted version for immune-therapeutics (iRECIST) were used according to the methods described by Eisenhauer *et al.* and Seymour *et al.* [16, 17]. A further description of these methods is given in Supplementary Methods, available at *Rheumatology* online.

### Statistics

The Fisher’s exact test was used for the comparison of proportions. The Mann–Whitney test was used for the comparison of continuous variables in two groups. In case more than two groups were compared, the Mann–Whitney *U* test was preceded by the Kruskal–Wallis test. The difference between the time to glucocorticoid free remission curves in the Kaplan–Meier graph was calculated by the log-rank test. Analyses were performed with GraphPad Prism 9. *P*-values <0.05 were considered statistically significant.

## Results

### Patient characteristics

A total of 37 patients with primary PMR and 15 patients with ICI-PMR were included in the main study analyses. Most patients with ICI-PMR received ICI therapy for melanoma, adenocarcinoma of the lung or renal cell carcinoma (Supplementary Table S1, available at *Rheumatology* online). ICI therapy was administered as adjuvant therapy in one patient, whereas it was used for metastatic disease in all other cases. Nivolumab was administered to six patients, pembrolizumab to five patients and atezolizumab to two patients. Furthermore, two patients received a combination of nivolumab/ipilimumab. Other irAEs were observed in 10 (67%) patients with ICI-PMR (Supplementary Table S2, available at *Rheumatology* online), with some of these patients suffering from multiple, other complications. None of the patients received another anti-cancer drug during their treatment with ICI therapy.

### Symptoms and laboratory markers

Primary PMR and ICI-PMR presented by bilateral shoulder pain in all patients; and nearly all patients experienced hip pain or stiffness (Table 1). The primary PMR group experienced morning stiffness and weight loss more frequently than the ICI-PMR group. Considering the laboratory results, the number of thrombocytes and the proportion of patients with elevated ESR were higher in the primary PMR group. Nevertheless, 13 (86.7%) patients with ICI-PMR had at least an elevated ESR or CRP level. Patients with ICI-PMR less frequently fulfilled the EULAR/ACR and Chuang classification criteria for PMR than patients with primary PMR.

**Table 1.** Overview of symptoms and laboratory results

	ICI-PMR	Primary PMR	
	N = 15	N = 37	*P* value
Male gender, *n* (%)	9 (60.0)	16 (43.2)	0.362
Age, median (range)	71 (54.0–85.0)	73 (54.0–85.0)	0.386
Symptoms, *n* (%)			
Bilateral shoulder pain	15 (100.0)	37 (100.0)	>0.999
Normal RF/ACPA^a^	13 (92.4)	36 (97.3)	0.487
Hip pain or stiffness	13 (86.7)	34 (91.9)	0.619
No involvement of other joints	7 (46.7)	26 (70.3)	0.126
Morning stiffness for >45 min	10 (66.7)	34 (91.9)	0.036*
Fullfilling classification criteria for PMR, *n* (%)			
EULAR/ACR criteria	10 (66.7)	36 (97.3)	0.006*
Chuang criteria	5 (33.3)	31 (83.8)	0.001*
Other symptoms present, *n* (%)			
Weight loss	1 (6.7)	21 (56.8)	0.001*
Fever	0 (0.0)	7 (18.9)	0.093
Back pain	3 (20.0)	9 (24.3)	>0.999
Cranial symptoms	3 (20.0)	9 (24.3)	>0.999
Number of days until, median (range)^b^			
Start of symptoms of PMR	86 (1.0–595.0)	—	—
Diagnosis of PMR	152 (40.0–731.0)	—	—
Number of ICI-cycles until, median (range)			
Start of symptoms of PMR	4 (1.0–27.0)	—	—
Diagnosis of PMR	7 (1.0–31.0)	—	—
Laboratory results, median (range)^c^			
Haemoglobin in g/L	7.6 (5.2–9.7)	7.7 (5.6–9.3)	0.708
Thrombocyte count in 10^9^/L	273 (105.0–448.0)	330 (170.0–562.0)	0.027*
CRP in mg/L	19 (0.4–91.0)	34 (3.2–186.0)	0.052
ESR in mm/h	35 (9.0–93.0)	53 (7.0–109.0)	0.113
Elevated lab results, *n* (%)			
CRP elevated^d^	11 (73.3)	34 (91.9)	0.173
ESR elevated^e^	8 (61.5)	33 (89.2)	0.040*

a RF/ACPA was measured in 14 patients with ICI-PMR.

b Days are counted from the start of ICI therapy.

c Three ICI-PMR patients received oral glucocorticoid treatment prior to the blood tests, of which two received hydrocortisone because of hypophysitis.

d Elevated if CRP >5 mg/L.

e ESR elevated if >20 mm/h in men and >30 mm/h in women; ESR was measured in 13 patients with ICI-PMR.

ACPA: anti-citrullinated protein antibody; CRP: C-reactive protein; ESR: erythrocyte sedimentation rate; RF: rheumatoid factor. *P value < 0.05.

### Ultrasound

Ultrasonography of the shoulders was performed in 14 ICI-PMR cases, of which 10 patients also received ultrasound scans of the hips. Only seven of the 37 patients with primary PMR from the main study cohort underwent shoulder and hip ultrasonography. Therefore, this group was supplemented with data from 16 additional, consecutive PMR patients that underwent ultrasonography of shoulders and hips. This supplementary group of patients with primary PMR was comparable to the patients in the main study analyses, except for weight loss ,which was less often observed in these additional patients (Supplementary Table S3, available at *Rheumatology* online). Among patients with ICI-PMR and primary PMR, bicep tenosynovitis and subacromial bursitis were the most prevalent abnormalities, while glenohumeral synovitis was least prevalent. There were no statistically significant differences between the groups (Table 2).

**Table 2.** Ultrasonography results

	ICI-PMR	Primary PMR	*P* value
Received ultrasound of the shoulders, *n*/total *n*	14/15	23/53	
Shoulder abnormality detected by ultrasound, *n* (%)			
Bicep tenosynovitis			
Bilateral	6 (42.9)	8 (34.8)	0.732
At least unilateral	12 (85.7)	13 (56.5)	0.084
Subacromial bursitis			
Bilateral	3 (21.4)	7 (30.4)	0.710
At least unilateral	7 (50.0)	14 (60.9)	0.733
Glenohumeral synovitis			
Bilateral	1 (7.1)	0 (0.0)	0.378
At least unilateral	3 (21.4)	6 (26.1)	>0.999
Received ultrasound of the hips, *n*/total *n*	10/15	23/53	
Hip abnormality detected by ultrasound, *n* (%)			
Trochanteric bursitis			
Bilateral	1 (10.0)	8 (34.8)	0.217
At least unilateral	4 (40.0)	11(47.8)	0.722
Hip synovitis			
Bilateral	2 (20.0)	5 (21.7)	>0.999
At least unilateral	4 (40.0)	10 (43.5)	>0.999
Sum of abnormalities on ultrasound, median (range)^a^	4 (1.0–5.0)	3 (0.0–8.0)	0.835
EULAR/ACR criteria fulfilled, *n* (%)^b^			
At least one shoulder AND hip	6 (60.0)	12 (52.2)	0.722
Bilateral shoulder involvement	4 (40.0)	12 (52.2)	0.708
EULAR/ACR score on ultrasound, median (range)	1 (0.0–2.0)	1 (0.0–2.0)	0.615

a Each abnormality was given 1 point, bilateral abnormalities were given 2 points. Consequently, the range of points to be given is 0–10. The median and range over the sum was calculated for the 10 patients with ICI-PMR that received ultrasound scans of both the shoulders and hips, as well as for all the patients with primary PMR.

b For each of the criteria one point is given, making the maximum score two points.

### ^18^F-FDG-PET uptake

**Figure 1.**
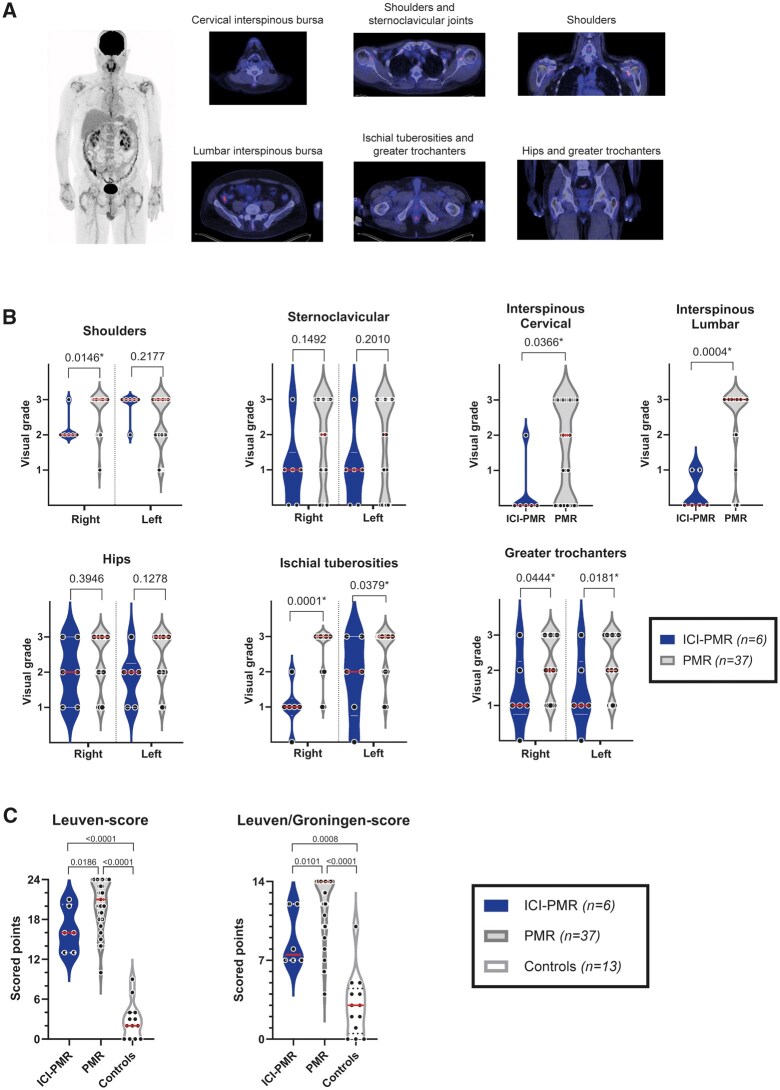
^18^F-FDG-PET/CT in patients with ICI-PMR and primary PMR. (**A**) Mean intensity projection of ^18^F-FDG-uptake (left panel) and fusion ^18^F-FDG-PET/CT images (right panel) of key inflammatory sites in a patient with ICI-PMR. (**B**) Visual grading of ^18^F-FDG-uptake at the inflammatory sites in patients with ICI-PMR (*n* = 6) and primary PMR (*n* = 37). Visual grading is performed as: 0, no uptake; 1, uptake < liver; 2, uptake = liver; and 3, uptake > liver. Middle lines indicate the median, while dotted lines indicate quartiles. (**C**) Leuven (left panel) and Leuven/Groningen (right panel) ^18^F-FDG-PET/CT scores in the same patients as mentioned at (B) and in 13 controls without an inflammatory rheumatic condition. Statistical significance by Mann–Whitney test is indicated above the plots

An ^18^F-FDG-PET/CT scan at the time of diagnosis of PMR was available for six patients with ICI-PMR and 37 patients with primary PMR. Statistically significant differences between the groups were observed in both the spinal regions, greater trochanters and ischial tuberosities, with lower ^18^F-FDG-uptake in the ICI-PMR group (Fig. 1). The Leuven and the Leuven/Groningen scores were significantly lower in the ICI-PMR group. Nevertheless, these ^18^F-FDG-PET/CT scores were still higher in patients with ICI-PMR compared with control patients without a rheumatic inflammatory disease (Fig. 1). Among patients with ICI-PMR, ^18^F-FDG-PET/CT scans performed prior to initiation of ICI therapy did not show increased ^18^F-FDG-uptake in comparison to the controls (Supplemental Fig. S1, available at *Rheumatology* online).

### Treatment

Treatment requirements for PMR were evaluated in the 15 patients with ICI-PMR and the 37 patients with primary PMR from the main cohort. Four patients with ICI-PMR (27%) could be managed without any oral glucocorticoid treatment. Two of these patients received an intramuscular depot and/or local injection of glucocorticoids, whereas the other two patients received no glucocorticoids at all for their ICI-PMR. Among the 37 patients with primary PMR, three patients were excluded from the analysis due to limited follow-up data. Four out of 37 patients (10.8%) were not treated with oral glucocorticoids: two patients were managed with intramuscular injections of glucocorticoids; and two patients refused any glucocorticoid treatment.

**Figure 2.**
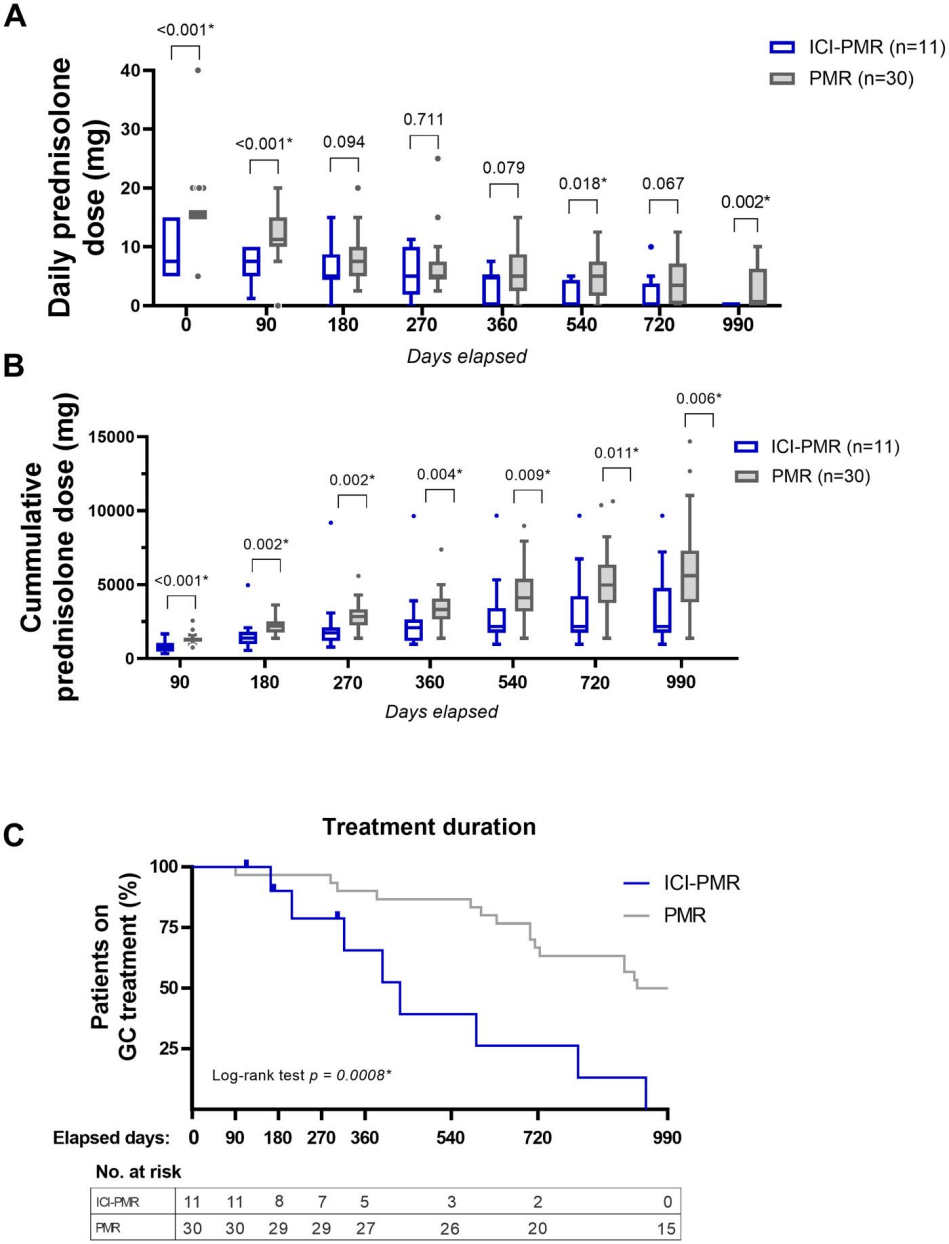
Treatment of ICI-PMR and primary PMR. Data is shown for 11 patients with ICI-PMR and 30 patients with primary PMR, unless stated otherwise. Asterisk indicates a statistically significant difference (*P* < 0.05). (**A**) Tukey plots of oral glucocorticoid dose (prednisolone equivalent) per time point. Statistical significance by Mann–Whitney test is indicated above the plots. (**B**) Tukey plots of cumulative oral glucocorticoid dose (prednisolone equivalent) per time point. Any oral glucocorticoid dose given for ICI-PMR or other irAEs was included in the analysis. Statistical significance by Mann–Whitney test is indicated above the plots. (**C**) Kaplan–Meier curve showing oral glucocorticoid treatment duration in patients with ICI-PMR and primary PMR. Only the treatment duration of ICI-PMR was taken into account (i.e., treatment for other irAEs was not included in the analysis). Statistical significance by the log-rank test is shown

Next, glucocorticoid requirements were evaluated in the remaining patients with ICI-PMR (*n* = 11) and primary PMR (*n* = 30), who had been monitored after initiation of oral glucocorticoid treatment. The median prednisolone starting dose (or equivalent glucocorticoid dose) in the primary PMR group was 15 mg/day, and 7.5 mg/day in the ICI-PMR group. The effective prednisolone dose (i.e., the highest dose necessary to induce a remission) did not exceed 10 mg/day in seven out of 11 patients with ICI-PMR (64%). Moreover, the four patients receiving prednisolone >10 mg/day (max. 15 mg/day) did so for a relatively short period, namely: 2 days, 16 days, 24 days and 30 days, respectively. The height of the prednisolone dose at most time points during follow-up was typically lower in the ICI-PMR group, when compared with the primary PMR group (Fig. 2A). Consequently, patients with ICI-PMR received a lower cumulative glucocorticoid dose than those with primary PMR (Fig. 2B).

Successful tapering (i.e., decreasing the glucocorticoid dose without the need to increase the dose in the first 30 days thereafter) occurred earlier in the ICI-PMR group (Supplementary Table S4, available at *Rheumatology* online). The glucocorticoid treatment duration of the ICI-PMR group (median 317 days, range 113–945 days) was significantly shorter than the treatment duration of the primary PMR group (median 963 days, range 91–990 days). Overall, patients with ICI-PMR showed less need for prolonged glucocorticoid treatment than patients with primary PMR, as indicated by the Kaplan–Meier curve (Fig. 2C). This lower treatment requirement in ICI-PMR was observed irrespective of the concomitant use of immunosuppressive therapy for other irAEs in part of patients (Supplementary Fig. S2, available at *Rheumatology* online). Among patients with ICI-PMR that could stop glucocorticoid treatment, withdrawal of glucocorticoid treatment was achieved both before and after ICI therapy discontinuation.

Disease-modifying antirheumatic drugs (DMARDs) were given to eight out of 37 patients (21.6%) of the primary PMR group. All of these patients received methotrexate, with two of these patients eventually switching to leflunomide. In comparison, one out of 15 patients with ICI-PMR (6.7%) received methotrexate (Supplementary Table S5, available at *Rheumatology* online).

### ICI therapy use after ICI-PMR diagnosis

Next, we evaluated how ICI-PMR diagnosis impacted ICI therapy decisions (Supplementary Table S6, available at *Rheumatology* online). At time of ICI-PMR diagnosis, ICI therapy was continued without any delay in eight out of 15 patients (53.3%). In two patients (13.4%), ICI therapy was continued after only a brief delay. In the remaining five patients (33.3%) ICI either had already been stopped for other reasons or was eventually stopped at least partly due to PMR. Patients in whom ICI therapy was continued (with or without a brief delay) showed a similar glucocorticoid requirement as patients in whom ICI therapy had already been stopped or was stopped at time of ICI-PMR diagnosis (Supplementary Fig. S3, available at *Rheumatology* online).

### Anti-tumour efficacy of ICI therapy

The overall tumour response (i.e., from the start until the end of ICI therapy) could be determined in 13 of the 15 ICI-PMR patients. One patient received adjuvant ICI therapy and could therefore not be evaluated by RECIST, while the other patient received just one cycle of ICI therapy. Of the 13 patients that could be evaluated, four patients (31%) had a complete response (CR); four patients (31%) had a partial response (PR); two patients (15%) had stable disease (SD); and three patients (23%) had progressive disease (PD) (Supplementary Table S7 and Supplementary Fig. S4, available at *Rheumatology* online).

## Discussion

This is the first study to directly compare ICI-PMR to primary PMR. Although most of the clinical findings and all ultrasonographic findings were comparable in both diseases, our laboratory and ^18^F-FDG-PET/CT assessments suggested that ICI-PMR is associated with less intense inflammation than primary PMR. This was further substantiated by the milder disease course and lower treatment requirement observed in the ICI-PMR group.

Our findings indicate that a step-up approach (i.e., starting with a prednisolone dose <10 mg/day and only increasing the dose when necessary) is an excellent treatment strategy for ICI-PMR. The majority of patients with ICI-PMR required low prednisolone doses ≤10 mg. Only four out of 15 patients required a daily dose of prednisolone >10 mg (max. 15 mg), for less than a month. These results are in strong contrast with previous studies concluding that ICI-PMR requires high prednisolone doses (e.g., up to 60 mg/day) [8, 9]. Only one out of 15 patients (6.7%) in our ICI-PMR cohort required a DMARD in addition to glucocorticoid therapy. In a prior case series, four out of 20 patients (25%) required treatment with biological (e.g., tocilizumab) or conventional synthetic (e.g., methotrexate or hydroxychloroquine) DMARDs [8]. The reason for these difference remains unclear. It could be, as Calabrese *et al.* stated, that high doses were initially given due to a lack of experience and confidence of the treating physicians [8]. Furthermore, the higher doses were seen predominantly in one out of three healthcare centres, suggesting a regional influence on treatment approach. Ideally, ICI-PMR is treated with as little immunosuppressive therapy as possible, with ‘EULAR points to consider’ suggesting not to exceed a prednisolone dose of 10 mg/day due to potential negative effects on the anti-tumour efficacy of ICI therapy [18]. Bearing in mind the importance of preserving anti-tumour efficacy of ICI therapy, our study provides reassuring insight that mild immunosuppressive treatment might suffice for the management of ICI-PMR.

ICI-PMR requires a shorter treatment duration than primary PMR. Successful tapering of prednisolone treatment occurred earlier in the ICI-mediated PMR group than in the primary PMR group. In theory, this could have been influenced by differences in treatment strategies between these groups. For example, the prednisolone starting dose was lower in the ICI-PMR group and tapering was attempted earlier than in the primary PMR group. However, the majority of our patients with primary PMR still required prednisolone treatment beyond two years, which is in accordance with a prior report by Mackie *et al.* [19]. Because standard tapering protocols for primary PMR would have allowed for prednisolone treatment to be stopped much earlier, this indicates that the prolonged treatment in patients with primary PMR is actually necessary due to frequently occurring relapses. Overall, our study indicates that ICI-PMR really has a shorter treatment requirement than primary PMR.

Our study indicates that ICI therapy can usually be continued when ICI-PMR occurs. The ICI therapy was not interrupted, or delayed for a very short period only, when our patients received a diagnosis of ICI-PMR. ICI-PMR could thus be effectively managed despite continuation of ICI therapy, as also observed in other reports [4, 9]. This is important as ICI therapy might be critical for the survival of patients with cancer.

Our clinical, laboratory and imaging assessments suggest that ICI-PMR is characterized by less intense inflammation than primary PMR. The ICI-PMR group less often showed morning stiffness, weight loss and elevation of the ESR. Although ultrasonographic findings were comparable in ICI-PMR and primary PMR, the metabolic activity of the inflammatory lesions detected on ^18^F-FDG-PET/CT was lower in the ICI-PMR group. Previous studies already hinted that ICI-PMR often presents with atypical features [8, 10]. A possible explanation for the different intensity of inflammation could be that the underlying, immunological perturbations vary between ICI-PMR and primary PMR. Based on ICI therapy’s mode of action, we suspect that inflammation in ICI-PMR is mainly caused by infiltrating T cells, whereas macrophages appear to be the key mediator of inflammation in primary PMR [20, 21]. This hypothesis requires confirmation by translational immunology studies focussing on ICI-PMR.

In our study, it was reassuring that the majority of patients with ICI-PMR showed a tumour response to ICI therapy. Among the 13 patients with sufficient oncologic data available, we observed complete remission (*n* = 4), partial remission (*n* = 4) or stable disease (*n* = 2). Only three patients showed progressive disease. Several studies have shown that the presence of irAEs associates with a more favourable overall survival and response to ICI therapy [4, 22]. However, our findings on the anti-tumour efficacy of ICI therapy should be interpreted with caution, given the small sample size and oncological heterogeneity of our patients.

In accordance with prior studies, our study suggests that ICI-PMR is a PMR-like syndrome rather than a true form of primary PMR. Given the tendency of ICI-PMR to present with atypical features (e.g., the absence of morning stiffness or low inflammation markers in blood), imaging confirmation might be needed to justify the use of immunosuppressive therapy in patients receiving ICI therapy. We here propose that the diagnosis of ICI-PMR should be based on the presence of pain/stiffness in the shoulder and hip girdle in association with detection of inflammatory lesions at these sites by imaging.

Our study has strengths and limitations. A strength of this study is the direct comparison of the clinical, laboratory, imaging and treatment characteristics of ICI-PMR and primary PMR. A limitation of the study is its retrospective design. Therefore, we were confined to what was documented and exposed to potential biases in this documentation. There is a risk of selection bias, because this study has been conducted in a single healthcare centre; and because ICI-PMR patients were only included when they were referred to the rheumatology department. Patients with less severe complaints might have been treated by oncologists themselves, without our knowledge. Some patients used immunosuppressive therapy for other irAEs. However, we found that this additional treatment did not explain the low glucocorticoid treatment in patients with ICI-PMR. The number of patients with ICI-PMR was relatively limited. Nevertheless, it represents the largest single-centre report systematically describing disease characteristics of ICI-PMR.

## Conclusion

Our findings support the notion that ICI-PMR is a different disease entity than primary PMR. Although ICI-PMR and primary PMR share a cluster of symptoms related to inflammation in the shoulder and hip girdle, ICI-PMR is associated with less intense inflammation and a lower treatment requirement than primary PMR. Although subsequent studies are necessary, our findings provide a framework for the diagnostic and therapeutic approach to this new PMR-like syndrome.

## Supplementary material

Supplementary material is available at *Rheumatology* online.

## Supplementary Material

keae099_Supplementary_Data

## Data Availability

Data underlying this article are available in the article and in its online supplementary material.
