# Supplementary material for: Immune checkpoint inhibitor-mediated polymyalgia rheumatica versus primary polymyalgia rheumatica: comparison of disease characteristics and treatment requirement
Source: Rheumatology (Oxford). 2024 Feb 9;64(2):771–9. doi: 10.1093/rheumatology/keae099 (PMC11781573; doi:10.1093/rheumatology/keae099)
Supplement: keae099_Supplementary_Data [file keae099_supplementary_data.docx]

# Supplementary Methods

## ^18^F-FDG-PET/CT

^18^F-FDG-PET/CT scanning was performed according to the European Association of Nuclear Medicine Guidelines (1,2). The scans were acquired from the vertex to the knees on an integrated PET/CT system, specifically the Biograph CT 40 or 64 slice PET/CT (Siemens, Knoxville, TN, USA) or the Biograph Vision PET/CT, with a setting of 3 min per bed position. Prior to the acquisition, patients fasted for a minimum of six hours, blood glucose was measured and 3 MBq intravenous ^18^F-FDG was administered per kg of bodyweight. Approximately one hour later the scan was performed. For anatomic mapping and attenuation correction a low-dose CT scan was used (100kV and 30mAs) .

## RECIST 1.1 and iRECIST evaluation methods

Tumour response was measured by using the Response Evaluation Criteria in Solid Tumours 1.1 (RECIST 1.1) as well as the adapted version for immune-therapeutics (iRECIST); according to the methods described by *Eisenhauer et al.* and *Seymour et al.* (3,4). Evaluation of the tumour response in the ICI-group was conducted under the supervision of an experienced oncologist (GH).

With these methods the tumour growth or decline was measured using CT-scan imaging. First, a baseline CT-scan is made on which a maximum of 5 easy to measure target lesions are chosen. Non-target lesions are identified next, these are lesions not suited for exact measurement, for example ascites. The sum of the longest diameters (SLD) of the target lesions is calculated. On subsequent scans the SLD is compared and the development of non-target lesion is determined. With this information the response can be determined. The four types of responses are: 1) Complete Remission (CR): all lesions have disappeared, and pathologic lymph nodes have returned to normal sizes (i.e., smaller than 10 mm in the short axis), 2) Partial Response (PR): the SLD has decreased with more or equal to 30% of the previous scan. Furthermore, there can be no new lesions and no progression of non-target lesions. 3) Stable Disease (SD): there is no progressive disease and no partial or complete response. 4) Progressive Disease (PD): there is an increase of equal or more than 20% of the SLD, when compared to the smallest SLD in the study. Furthermore, there is PD when there is progression of non-target lesions or when new lesions have formed. As for the iRECIST criteria, PD responses are further subdivided into confirmed progressive disease (iCPD) and unconfirmed progressive disease (iUPD). In short, the difference is based on whether the subsequent scan shows further sings of disease progression. If there is continued progression the label iCPD is given, and if there is no further progression the response is labelled as iUPD.

In case of inadequate CT-scans, response was evaluated clinically based on ^18^F-FDG-PET scans, MRI scans and clinical descriptions. In these cases the response was measured only by RECIST 1.1. Tumour response was measured for three intervals: an overall response during ICI therapy (i.e., the response between the start and end of ICI therapy); before GCs (i.e., the response between the start of ICI therapy until the start of GCs for ICI-PMR); and during GCs (i.e., the response between the start and end of GCs for ICI-PMR).

# Supplementary Tables

**Supplementary Table S1. Characteristics of the ICI-mediated PMR (ICI-PMR) group.**

|  | ICI-PMR |
| --- | --- |
|  | **N = 15** |
| Type of cancer, n (%) |  |
| Melanoma | 5 (33.3) |
| Adenocarcinoma of the lung | 3 (20.0) |
| Renal cell carcinoma | 2 (13.3) |
| Urothelial carcinoma of the bladder | 1 (6.7) |
| Squamous cell carcinoma of the lung | 1 (6.7) |
| Cervix carcinoma | 1 (6.7) |
| Pancreas carcinoma | 1 (6.7) |
| Mesothelioma | 1 (6.7) |
|  |  |
| Metastasized, n (%)^a^ | 14 (93.3) |
|  |  |
| Type of ICI therapy, n (%) |  |
| Nivolumab | 6 (40.0) |
| Pembrolizumab | 5 (33.3) |
| Nivolumab/ipilimumab | 2 (13.3) |
| Atezolizumab | 2 (13.3) |
|  |  |
| Number of ICI therapy cycles received, median (range) |  |
| At start of symptoms PMR | 4 (1.0-27.0) |
| At diagnosis of PMR | 7 (1.0-31.0) |
| Total | 14 (1.0.-51.0) |
| Number of days since start ICI therapy, median (range) |  |
| Start of PMR symptoms | 86 (1.0-595.0) |
| Diagnosis of PMR | 152 (40.0-731.0) |
| *^a^ In the patient with non-metastasized cancer, ICI-therapy was given as adjuvant therapy.* | |

**Supplementary Table S2. Overview of other immune-related adverse events (irAEs) occurring in patients with ICI-PMR.**

|  | ICI-PMR |
| --- | --- |
|  | **N=15** |
| Type of IrAE, n (%) |  |
| Thyroiditis | 3 (20.0) |
| Hypophysitis | 2 (13.3) |
| Keratoconjunctivitis | 2 (13.3) |
| Hepatitis | 2 (13.3) |
| Colitis | 1 (6.7) |
| Mucocutaneous pemphigoid | 1 (6.7) |
| Pneumonitis | 1 (6.7) |
| None | 5 (33.3) |
|  |  |
| Number of cycles at time of onset irAE, median (range) | 7 (1.0-14.0) |
|  |  |
| Received immunosuppressive medication for other IrAE at diagnosis ICI-PMR, n (%) | 3 (20) |
| Type of immunosuppressive medication, n (%)^a^ |  |
| Budenofalk for colitis | 1 (6.7) |
| Hydrocortison for hypocortisolism | 2 (13.3) |
|  |  |
| Received immunosuppressive medication for other IrAE after diagnosis ICI-PMR | 4 (26.7) |
| Budenofalk, infliximab (1 infusion) and vedolizumab (4 infusions) for colitis | 1 (6.7) |
| Hydrocortison for hypocortisolism | 1 (6.7) |
| Dapsone for pemphigoid | 1 (6.7) |
| Cellcept (11 days) for pneumonitis and tacrolimus for hepatitis ^a^ | 1 (6.7) |
|  |  |
| ^a^ One patient received methylprednisolone for pneumonitis. | |

**Supplementary Table S3. Clinical data of the 16 additional patients with primary PMR undergoing ultrasonography.**

|  | Additional patients with primary PMR | Main cohort of patients with primary PMR |  |
| --- | --- | --- | --- |
|  | **N = 16** | **N = 37** | **p value** |
| Male gender, n (%) | 7 (43.8) | 16 (43.2) | >0.999 |
| Age, median (range) | 69 (56.0-79.0) | 73 (54.0-85.0) | 0.157 |
| Symptoms, n (%) |  |  |  |
| Bilateral shoulder pain | 16 (100.0) | 37 (100.0) | >0.999 |
| Normal RF/ACPA | 13 (81.3) | 36 (97.3) | 0.077 |
| Hip pain or stiffness | 16 (100.0) | 34 (91.9) | 0.545 |
| No involvement of other  joints | 14 (87.5) | 26 (70.3) | 0.299 |
| Morning stiffness for > 45 minutes | 13 (81.3) | 34 (91.9) | 0.351 |
| Fulfilling classification criteria for PMR, n (%) |  |  |  |
| EULAR/ACR criteria | 15 (93.8) | 36 (97.3) | 0.517 |
| Chuang criteria | 12 (75.0) | 31 (83.8) | 0.467 |
| Other symptoms, n (%) |  |  |  |
| Weight loss | 4 (25.0) | 21 (56.8) | 0.041* |
| Fever | 3 (18.8) | 7 (18.9) | >0.999 |
| Back pain | 5 (31.3) | 9 (24.3) | 0.736 |
| Cranial symptoms | 2 (12.5) | 9 (24.3) | 0.471 |
| Laboratory results, median (range) |  |  |  |
| Haemoglobin in g/L | 7.9 (7.2-9.4) | 7.7 (5.6-9.3) | 0.535 |
| Thrombocytes count in 10^9^/L | 340 (197.0-517.0) | 330.0 (170.0-562.0) | 0.334 |
| CRP in mg/L | 36.5 (2.7-89.0) | 34.0 (3.2-186.0) | 0.859 |
| ESR in mm/hr | 56 (22.0-80.0) | 53 (7.0-109.0) | 0.705 |
| Elevated lab results, n (%) |  |  |  |
| CRP elevated ^a^ | 15 (93.8) | 34 (91.9) | >0.999 |
| ESR elevated ^b^ | 14 (87.5) | 33 (89.2) | >0.999 |
| ^a^ *CRP elevated if CRP > 5mg/L* ^b^ *ESR elevated if ESR > 20 mm/hr in males and ESR > 30 mm/hr in females* | | | |

**Supplementary Table S4. Time of first successful tapering of glucocorticoid dose.** Successful tapering was defined as a decrease in the glucocorticoid dose without the need to increase the dose in the first 30 days thereafter.

|  | ICI-PMR | Primary PMR | P-value |
| --- | --- | --- | --- |
|  | **N = 11** | **N = 30** |  |
| Number of days, median (range) |  |  |  |
| Until first successful tapering | 25.0 (4.0-88.0) | 46.5 (17.0-186.0) | 0.029* |

**Supplementary Table S5. Use of DMARDS for ICI-PMR or primary PMR.**

|  | ICI-PMR | Primary PMR |
| --- | --- | --- |
|  | **N = 15** | **N = 37** |
| Use of DMARDs, n (%) |  |  |
| Methotrexate | 1 (6.7) | 8 (21.6) |
| Leflunomide^a^ | 0 (0.0) | 2 (5.4) |
|  |  |  |
| Methotrexate use, median (range) |  |  |
| Start use methotrexate, in days after start GC | 69 | 378.5 (98.0-798.0) |
| Methotrexate treatment duration, in days | 57 | 319.5 (98.0-699.0) |
|  |  |  |
| Leflunomide use, median (range) |  |  |
| Start use Leflunomide, in days after start GC, |  | 254.5 (0.0-509.0) |
| Leflunomide treatment duration, in days |  | 221.0 (195.0-247.0) |
| ^a^ The two patients in the PMR group that received leflunomide treatment first received methotrexate treatment. | | |

**Supplementary Table S6. ICI therapy after diagnosis of ICI-PMR.**

|  | ICI-PMR |
| --- | --- |
|  | **N = 15** |
| PMR influence on ICI therapy, n (%) |  |
| Not delayed | 8 (53.3) |
| Temporarily delayed | 2 (13.4) |
| Stopped | 3 (20.0) |
| ICI had already ended^a^ | 2 (13.4) |
| ICI treatment delay due to PMR in weeks, median (range) | 1.5 (1.0-3.0) |
|  |  |
| Eventual Reason for stopping ICI therapy, n (%)^b^ |  |
| End of treatment | 4 (33.3) |
| PMR in combination with complete response | 1 (8.3) |
| PMR in combination with complete response and other toxicity | 1 (8.3) |
| PMR in combination with end of treatment | 1 (8.3) |
| Other toxicity | 2 (16.7) |
| Insufficient effect | 2 (16.7) |
| Second tumour, therefore start chemotherapy | 1 (8.3) |
| *^b^ In two patients ICI-therapy had already ended when PMR symptoms started. One of these patients received one cycle of ICI, but had to stop due to hepatitis. PMR symptoms started 189 days after this cycle. The other patient received 4 cycles as adjuvant therapy. In this case symptoms of PMR started 131 days after the first ICI cycle. ^b^ ICI-therapy was not yet stopped in 3 patients at last visit.* | |

**Supplementary Table S7. Overview of tumour response for individual patient with ICI-PMR.**

|  | Response during interval | |  |
| --- | --- | --- | --- |
| Patient | ***Start ICI therapy to start GC*** | ***Start GCs to stop GC*** |  |
| #1 | PR | CR |  |
| #2 | CR | CR |  |
| #3 | PD (iUPD)^f^ | PR |  |
| #4 | *Did not receive GC ^a^* | |  |
| #5 | PR | PR |  |
| #6^e^ | PD | PD |  |
| #7 | *Did not receive GC ^a^* | |  |
| #8 | SD | CR |  |
| #9^e^ | *Did not receive GC ^a^* | |  |
| #10 | PD (iCPD) ^g^ | PD (iUDP) ^h^ |  |
| #11^e^ | PD | PD |  |
| #12 | *Did not receive GC ^a^* | |  |
| #13 | *Evaluation was not possible ^b^* | |  |
| #14 | PR | *GC treatment has not stopped ^c^* |  |
| #15 | SD | *GC treatment has not stopped ^c^* |  |
| CR = complete response, PR = partial response, SD = stable disease, PD = progressive disease, iUPD = unconfirmed progressive disease, iCPD = confirmed progressive disease, GC = glucocorticoid therapy.  ^a^ In the four patients who did not receive GC therapy, the first two intervals could not be evaluated. Patient #9 received a short two week treatment because of pemphigoid.  ^b^ In this patients RECIST evaluation was not possible, because ICI therapy was given as adjuvant therapy ^c^ In two patients GC steroid use had not yet stopped and the last available CT-scan was used for the *start ICI to stop ICI* interval. Therefore the start GC to stop GC interval could not be evaluated. ^d^ In patients #14 and #15 ICI therapy had not yet ended. Therefore, the last available CT scan in the patient file was used for the evaluation of the response; making this a preliminary response. Another consequence is that iRECIST evaluation could not be done in the last interval for these patients. ^e^ Patients #6, #9 and #11 had to be evaluated clinically, therefore iRECIST method was not used in these patients.  ^f^ Subsequent scans showed a decrease of target and non-target lesions, no new lesions had formed. ^g^ Subsequent scan showed an increase in SLD of 12.5%, therefore iCPD ^h^ Subsequent scan showed no further increase of target and non-target lesions, as well as no new lesions. | | | |

# Supplementary Figures

**Supplementary Figure S1. ^18^F-FDG-PET/CT scores in patients with ICI-PMR before ICI therapy had started versus non-inflammatory controls.** The Leuven Score (left panel) and Leuven/Groningen Score (right panel) are shown. Middle lines indicate the median and dotted lines indicate quartiles. Statistical significance by Mann-Whitney test is indicated above the plots.


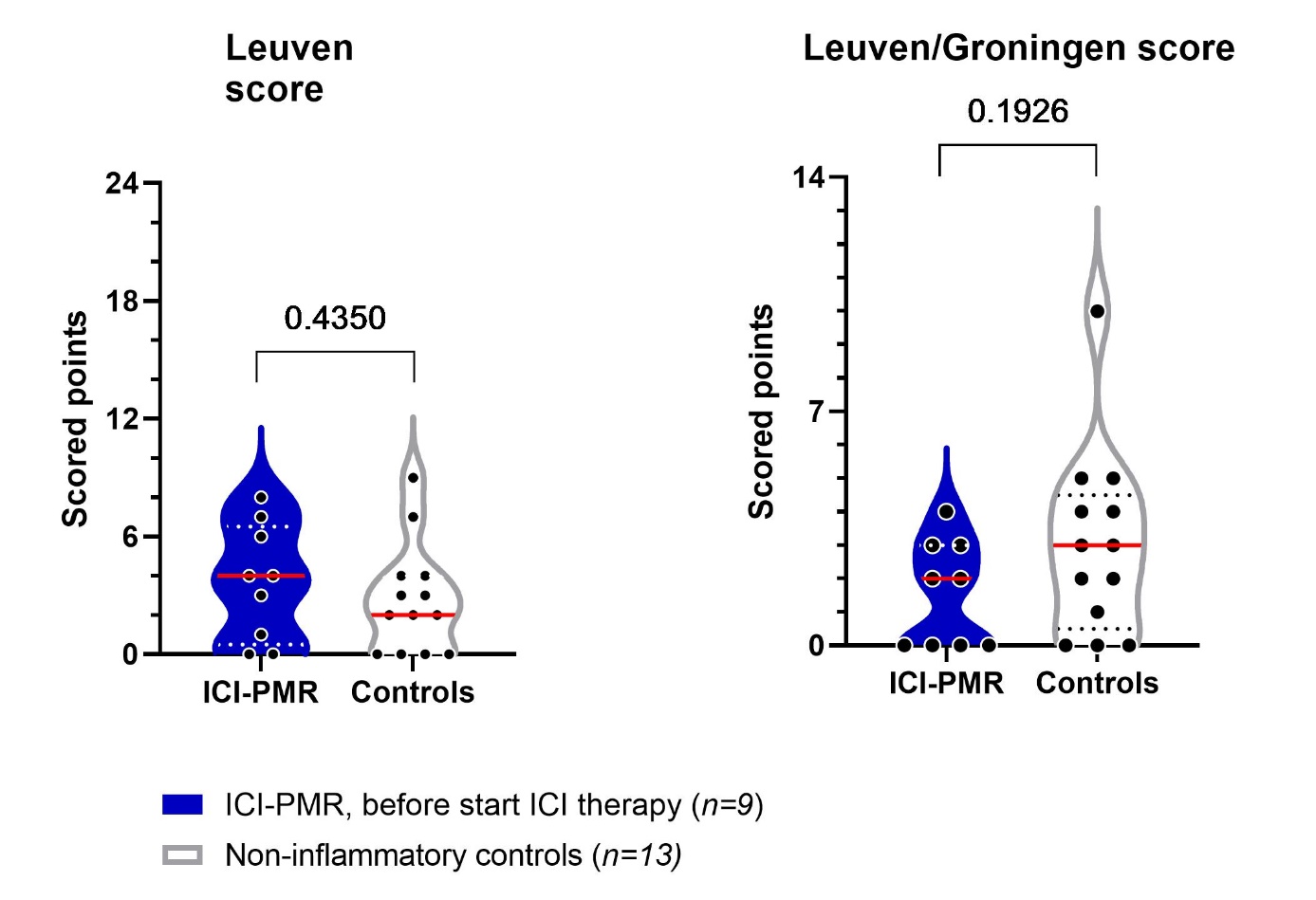


**Supplementary Figure S2. Kaplan-Meier curve showing oral glucocorticoid treatment duration in patients with ICI-PMR stratified for use of treatment for other irAEs.** Data are shown for the 11 patients requiring oral glucocorticoid therapy for ICI-PMR, and are compared to data for the 30 patients with primary PMR receiving oral glucocorticoid therapy. Among the four patients not requiring oral glucocorticoid therapy for ICI-PMR, one patient received additional immunosuppressive therapy for another irAE, whereas three patients received no other immunosuppressive therapy. Patients with ICI-PMR were stratified for use of concomitant immunosuppressive therapy for another irAE (‘other Tx’). Among patients with ICI-PMR, only the treatment duration of ICI-PMR was taken into account (i.e., treatment for the other irAEs was not included in the analysis). Statistical significance by the log-rank test is shown.


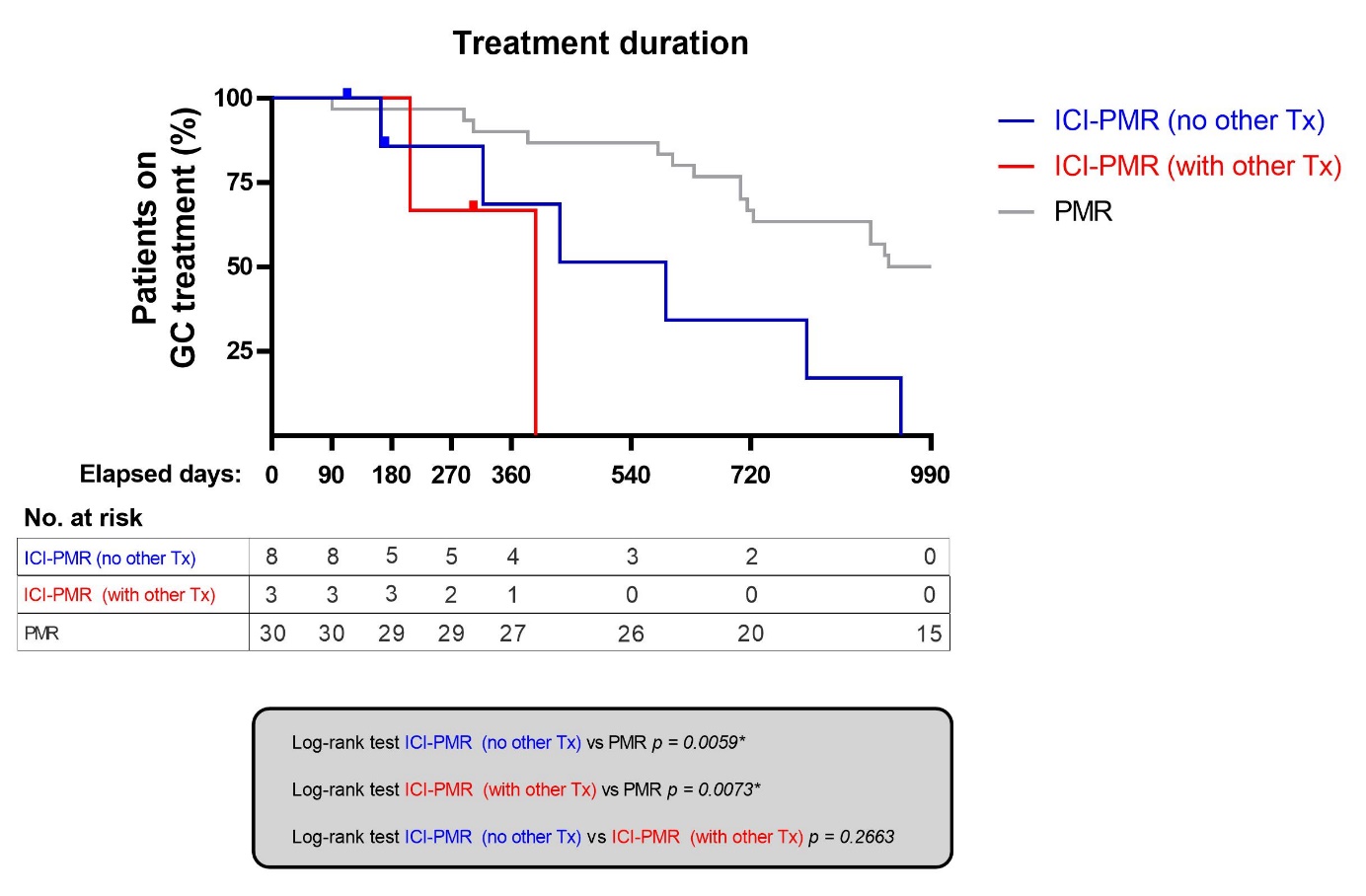


**Supplementary Figure S3. Kaplan-Meier curve showing oral glucocorticoid treatment duration in patients with ICI-PMR stratified for continuation or discontinuation of ICI therapy.** Data are shown for the 11 patients requiring oral glucocorticoid therapy for ICI-PMR. Among the four patients not requiring oral glucocorticoid therapy for ICI-PMR, ICI therapy was continued in three patients and stopped in one patient. Only the treatment duration of ICI-PMR was taken into account (i.e., treatment for the other irAEs was not included in the analysis). Patients with ICI-PMR were stratified for use of ICI therapy after the diagnosis of ICI-PMR: i.e. patients in whom ICI-therapy was continued (with/without a brief delay) versus patients in whom ICI-therapy had already been stopped for another reason or was eventually stopped partly due to ICI-PMR. Statistical significance by the log-rank test is shown.


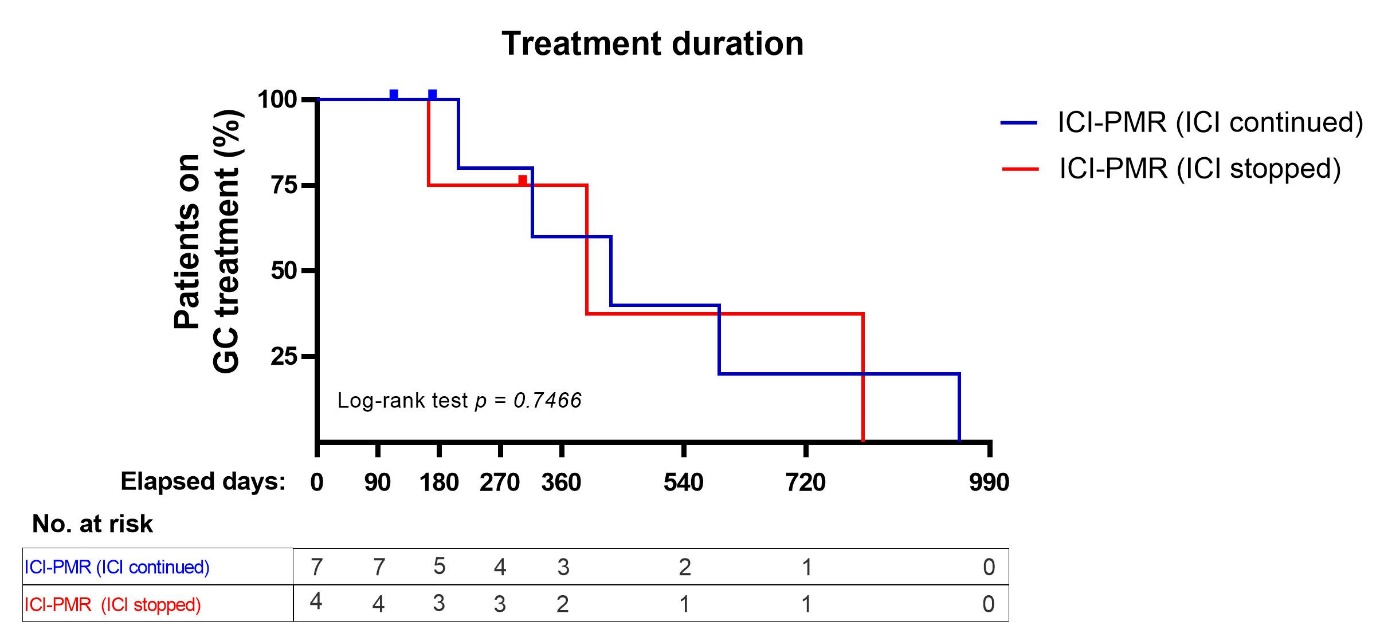


**Supplementary Figure S4. ICI and glucocorticoid treatment overview and the overall tumour response to ICI therapy.** The grey bar shows the number of days from the start of ICI therapy until the end of ICI therapy. The blue bar shows the number of days from the start until the end of glucocorticoid (GC) therapy. The time of diagnosis (i.e., the first time the treating rheumatologist mentioned ICI-PMR as diagnosis in the electronic patient file) is marked by the red line. On the right Y-axis the overall response to ICI therapy (i.e., the response from the start to the end of ICI therapy) is given. * ICI therapy was still ongoing at last visit.

**
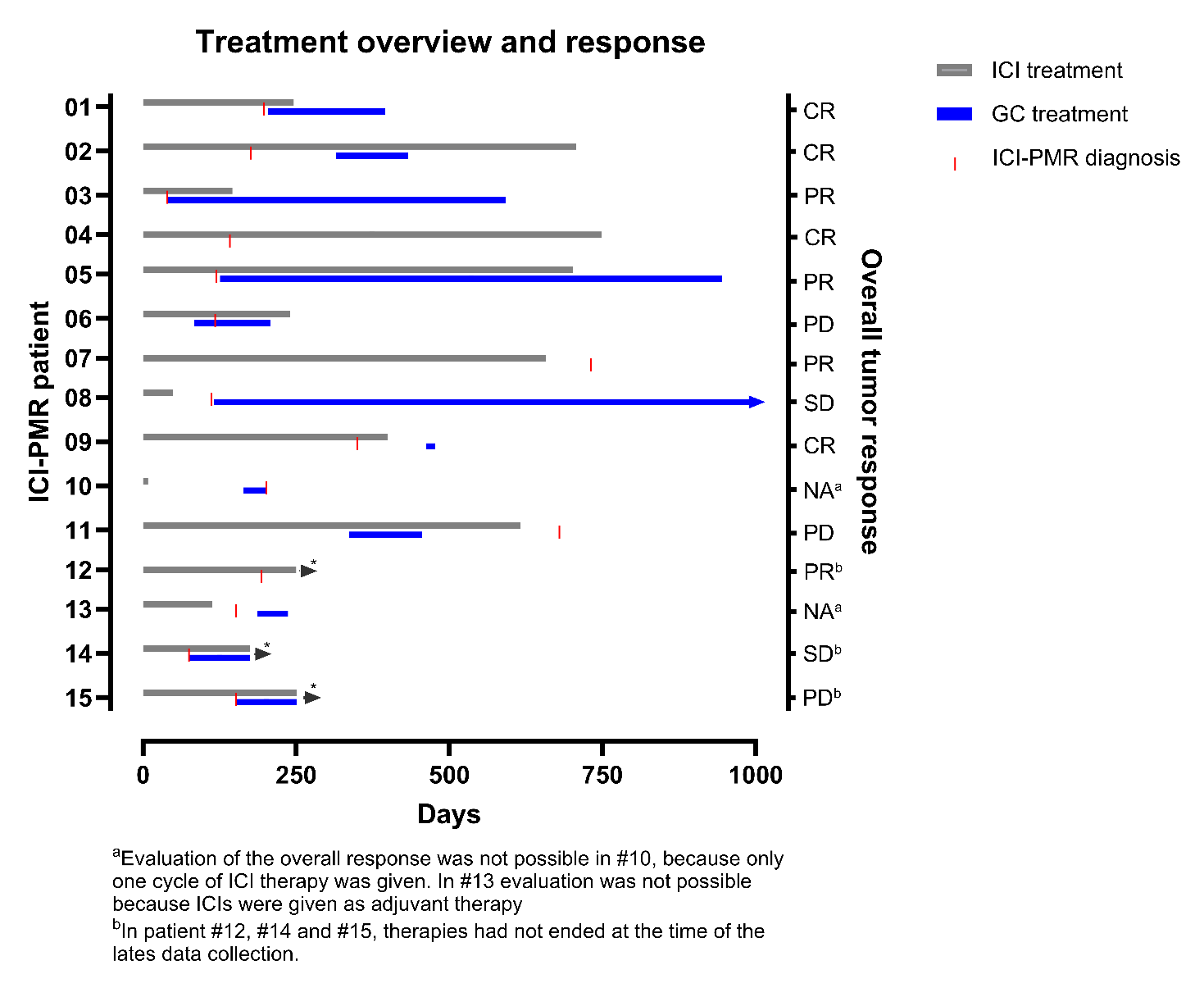
**

# References

1. Jamar F, Buscombe J, Chiti A, Christian PE, Delbeke D, Donohoe KJ, et al. EANM/SNMMI guideline for 18F-FDG use in inflammation and infection. Journal of Nuclear Medicine. 2013 Apr 1;54(4):647–58.

2. Slart RHJA, Slart RHJA, Glaudemans AWJM, Chareonthaitawee P, Treglia G, Besson FL, et al. FDG-PET/CT(A) imaging in large vessel vasculitis and polymyalgia rheumatica: joint procedural recommendation of the EANM, SNMMI, and the PET Interest Group (PIG), and endorsed by the ASNC. Vol. 45, European Journal of Nuclear Medicine and Molecular Imaging. Springer Berlin Heidelberg; 2018. p. 1250–69.

3. Eisenhauer EA, Therasse P, Bogaerts J, Schwartz LH, Sargent D, Ford R, et al. New response evaluation criteria in solid tumours: Revised RECIST guideline (version 1.1). Eur J Cancer. 2009 Jan;45(2):228–47.

4. Seymour L, Bogaerts J, Perrone A, Ford R, Schwartz LH, Mandrekar S, et al. Review iRECIST: guidelines for response criteria for use in trials testing immunotherapeutics [Internet]. www.thelancet.com/oncology. 2017. Available from: www.thelancet.com/oncology
